# Spin valley and giant quantum spin Hall gap of hydrofluorinated bismuth nanosheet

**DOI:** 10.1038/s41598-018-25478-6

**Published:** 2018-05-09

**Authors:** Heng Gao, Wei Wu, Tao Hu, Alessandro Stroppa, Xinran Wang, Baigeng Wang, Feng Miao, Wei Ren

**Affiliations:** 1 Department of Physics, Materials Genome Institute, Shanghai Key Laboratory of High Temperature Superconductors, and International Centre for Quantum and Molecular Structures, Shanghai University, 200444 Shanghai, China; 2 Consiglio Nazionale delle Ricerche (CNR-SPIN), Via Vetoio, I-67100 L’Aquila, Italy; 3 School of Electronic Science and Engineering, and Collaborative Innovation Center of Advanced Microstructures, Nanjing University, 210093 Nanjing, China; 4 National Laboratory of Solid State Microstructures, School of Physics, Collaborative Innovation Center of Advanced Microstructures, Nanjing University, 210093 Nanjing, China; 5 State Key Laboratory of Solidification Processing, Northwestern Polytechnical University, Xi’an, 710072 China

## Abstract

Spin-valley and electronic band topological properties have been extensively explored in quantum material science, yet their coexistence has rarely been realized in stoichiometric two-dimensional (2D) materials. We theoretically predict the quantum spin Hall effect (QSHE) in the hydrofluorinated bismuth (Bi_2_HF) nanosheet where the hydrogen (H) and fluorine (F) atoms are functionalized on opposite sides of bismuth (Bi) atomic monolayer. Such Bi_2_HF nanosheet is found to be a 2D topological insulator with a giant band gap of 0.97 eV which might host room temperature QSHE. The atomistic structure of Bi_2_HF nanosheet is noncentrosymmetric and the spontaneous polarization arises from the hydrofluorinated morphology. The phonon spectrum and *ab initio* molecular dynamic (AIMD) calculations reveal that the proposed Bi_2_HF nanosheet is dynamically and thermally stable. The inversion symmetry breaking together with spin-orbit coupling (SOC) leads to the coupling between spin and valley in Bi_2_HF nanosheet. The emerging valley-dependent properties and the interplay between intrinsic dipole and SOC are investigated using first-principles calculations combined with an effective Hamiltonian model. The topological invariant of the Bi_2_HF nanosheet is confirmed by using Wilson loop method and the calculated helical metallic edge states are shown to host QSHE. The Bi_2_HF nanosheet is therefore a promising platform to realize room temperature QSHE and valley spintronics.

## Introduction

The research of atomically thin two-dimensional (2D) materials has been a forefront topic in condensed matter physics since the successful exfoliation of single layer graphene^1^. A variety of new 2D systems have been proposed in theory and studied in experiment^2,3^ after the graphene discovery. 2D materials have sparked extensive investigation activities in recent years because of their rich physics and promising applications in optoelectronic devices and spintronics^4–7^. Furthermore, 2D materials can be the platforms to realize a variety of new quantum matter states. For example, 2D topological insulators (TIs)^8^, also known as quantum spin Hall (QSH) insulators, are a new state of quantum matter with an insulating bulk and metallic edge states which are protected by time-reversal symmetry against backscattering. As a matter of fact, graphene was the first proposed system to realize the quantum spin Hall effect (QSHE)^9^. However, the weak spin-orbit coupling (SOC) strength of carbon in graphene only induces a 10^−3^ meV bulk band gap which is too small to realize QSHE under ambient temperature^10^. The experimental realization of 2D TIs is so far limited to the HgTe/CdTe^11^ and InAs/GaSb^12^ quantum wells. Recently, a variety of large gap QSH insulators has been theoretically proposed. These large-gap 2D TIs include silicene^13^, 2D transition metal dichalcogenides^14^, III-Bi bilayers^15^, BiF 2D crystals^16^, Bi_4_Br_4_ 2D crystals^17^, ZrTe_5_ and HfTe_5_ 2D crystals^18^, tantalum carbide halides^19^, transition-metal carbides^20^, functionalized atomic lead films^21^ and transition-metal halide^22^. In experiments, the chemical functionalization or decoration is an efficient approach to modulate the physical properties of 2D materials. Compared with the zero-gap graphene, for instance, the hydrogenated or fluorinated graphene is an insulator^23,24^. In addition, there are several theoretical proposals for QSH insulators with large bulk band gap by decorating Bi-related monolayers using different chemical group^25–28^. However, to achieve room temperature functionality the theoretical and experimental searches of QSHE host with large bulk band gaps become critically important^29,30^.

For this purpose, we propose a hydrofluorinated Bi-based 2D structure namely Bi_2_HF nanosheet which intrinsically hosts topological band and valley properties. The codecoration of H and F atoms are on opposite sides of Bi buckled honeycomb monolayer. The electronegativity difference between the H and F atoms induces an intrinsic dipole moment which breaks the centrosymmetry in Bi_2_HF nanosheet. The Bi_2_HF nanosheet is dynamically and thermally stable, as confirmed by our following phonon and *ab initio* molecular dynamics calculations. The chemical functionalization with H and F will change electronic structures of the Bi 2D atomic layer. To figure out the coupling of spin and valley, we have used the first principles calculations and an effective Hamiltonian model to investigate the interplay between the intrinsic polarization and SOC. It shows an in-plane Rashba-type spin texture and an out-of-plane spin splitting of the band structure of Bi_2_HF nanosheet. Furthermore, we have also found that the Bi_2_HF nanosheet is a 2D topological insulator with a bulk band gap of 0.97 eV which can host room-temperature QSHE. Owing to the noncentrosymmetric structure of Bi_2_HF nanosheet, we used the Wilson loop method to confirm the topological invariant *Z*_2_ = 1. The helical metallic edge states of Bi_2_HF nanosheet are then revealed by recursive Green’s function approach based on Wannier basis Hamiltonian. The Bi_2_HF nanosheet is discovered as a promising platform to realize room temperature QSHE and valley spintronics.

## Results and Discussion

### Geometry, structural stability, and dipolar polarization

**Figure 1:**
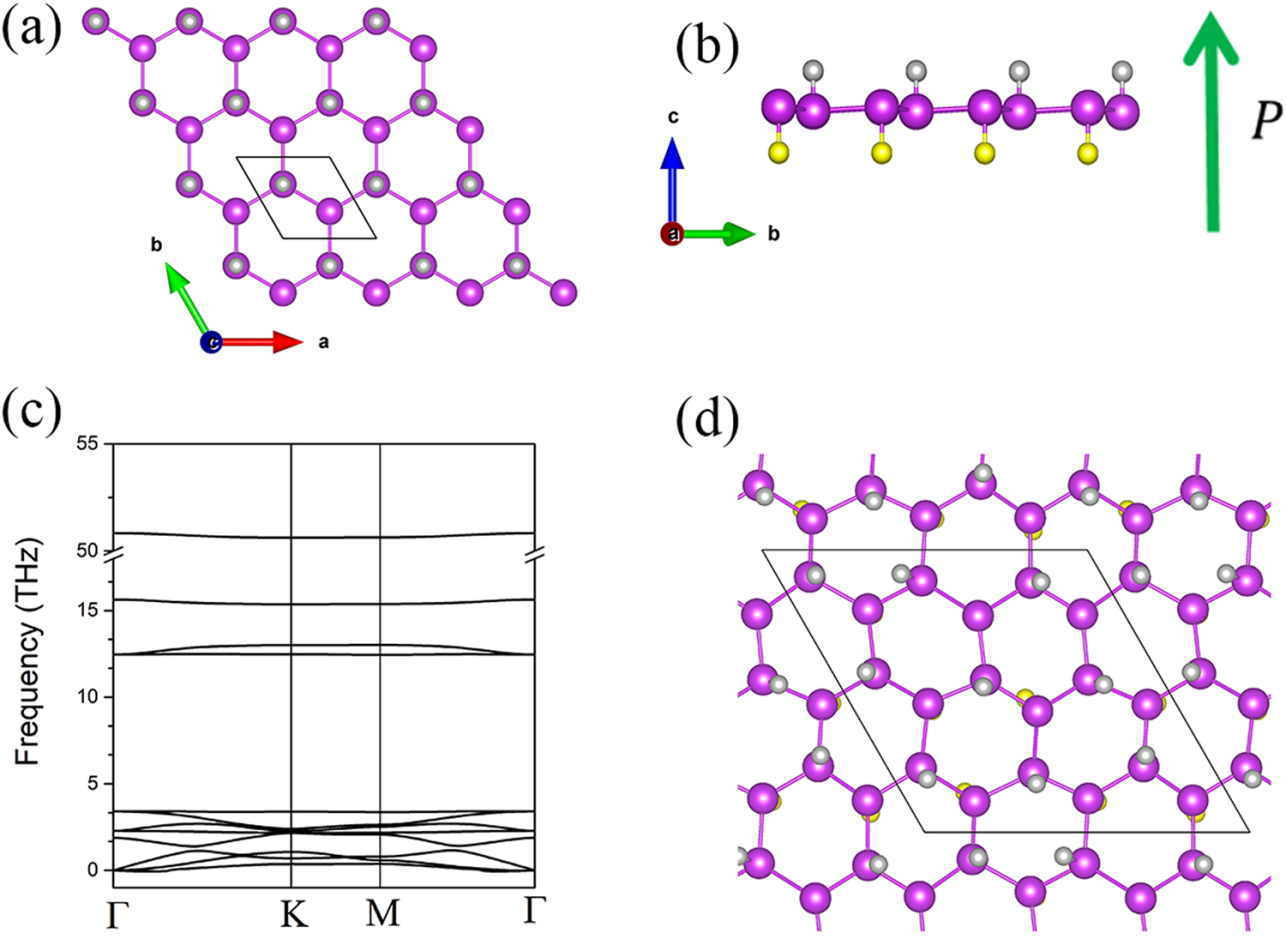
The atomistic structure of Bi_2_HF nanosheet (**a**) top view and (**b**) side view. The purple, gray and yellow balls denote Bi, H and F atoms, respectively. The big green arrow shows the direction of the intrinsic dipole polarization. (**c**) The phonon dispersion of Bi_2_HF nanosheet. (**d**) The snapshot from AIMD simulation of Bi_2_HF nanosheet at 300 K after 6 ps.

As shown in Fig. 1(a,b), the Bi_2_HF nanosheet is a 2D honeycomb lattice of buckled Bi atoms bonded with H and F atoms on opposite sides. The bond lengths of Bi-H and Bi-F are 1.84 and 2.08 Å, respectively. Such a functionalized structure of Bi_2_HF is similar to the hydrofluorinated graphene^31,32^. Unlike the Bi monolayer and fully hydrogenated or fluorinated Bi monolayer^27^, the structure of Bi_2_HF nanosheet is noncentrosymmetric owing to the H and F codecoration on opposite sides of Bi monolayer which breaks inversion symmetry and induces a net out-of-plane dipole moment. However, the switching of polarization directions without breaking chemical bonds is intrinsically impossible in this case. To compute and understand the dipole moment of Bi_2_HF nanosheet, starting from the centrosymmetric nonpolar fully hydrogenated Bi, we then substitute one by one of all H atoms on one side of the Bi bilayer with F atoms in a 2 × 2 × 1 supercell. The polarization of each structure is calculated using the Berry phase method^33^ and the results are shown in the Fig. S1. It turns out that the polarization of Bi_2_HF is 19.0 pC/m which is comparable to 18.5 pC/m of hydrogen and fluorine co-decorated silicene^34^. To study the relative stability of the Bi_2_HF nanosheet, the cohesive energy of Bi_2_HF nanosheet is calculated from the formula defined as

1 $${E}=\frac{1}{N}({{E}}_{{\rm{total}}}-{{n}}_{{\rm{Bi}}}{\mu }_{{\rm{Bi}}}-{{n}}_{{\rm{H}}}{\mu }_{{\rm{H}}}-{{n}}_{{\rm{F}}}{\mu }_{{\rm{F}}})$$

where *E*_total_ is the total energy of optimized system, *μ*_*i*_ (*i* = Bi, H, F) is the chemical potential of Bi, H and F, respectively. We obtain the chemical potential of Bi, H and F elements from bilayer Bi nanosheet, H_2_ gas, and F_2_ gas, respectively. The *n*_*i*_ (*i* = Bi, H, F) and *N* denote the numbers for different elements and total atomic number. The calculated cohesive energy value is found to be −5.35 eV suggesting that the Bi_2_HF is energetically stable and realizable in experiment. The cohesive energy of Bi_2_HF is comparable to −4.11 eV of hydrogen and fluorine co-decorated silicene^34^ and −6.56 eV of graphane^24^. We have also investigated other geometric configurations of Bi_2_HF and found that the chair configuration of Bi_2_HF shown in Fig. 1(a) has lower total energy than the boat and armchair configurations shown in Fig. S2. Thus we will focus on chair configuration in the following discussions.

To check the dynamic stability, the phonon dispersion of hydrofluorinated bismuth nanosheet has been calculated using the density functional perturbation theory (DFPT) approach^35^. The phonon band structure is shown in Fig. 1(c). There is no imaginary frequency observed in Brillouin zone (BZ) which means that the Bi_2_HF nanosheet is dynamically stable. We further confirm the thermodynamic stability of Bi_2_HF nanosheet by performing *ab initio* molecular dynamics (AIMD) calculation. A 3 × 3 supercell at temperature 300 K with a time step of 1 fs was studied. During the simulation of 6 ps at 300 K, no bond was broken in the honeycomb lattice, suggesting that the structure of Bi_2_HF nanosheet is thermally stable at room temperature. The snapshot from AIMD calculation of Bi_2_HF nanosheet at 300 K after 6 ps is shown in Fig. 1(d). One can see that there are no significant disruption or structural reconstruction in Bi_2_HF nanosheet at 300 K. Our theoretical simulations suggest that Bi_2_HF nanosheet is promising to be realized in experiment. Here we propose three steps to synthesize Bi_2_HF nanosheet based on the Bi (111) bilayer, which has been fabricated on the Bi_2_Te_3_ substrate in experiments^36^. In the first step, one can obtain half-fluorinated Bi bilayer by exposure to SF_6_ plasma^23^, then transfer half-fluorinated Bi nanosheet onto a new substrate attaching the fluorinated side. At last step, the hydrofluorinated bismuth nanosheet is achieved by exposing the other side surface to low-pressure hydrogen-argon mixture with dc plasma^37^.

### Electronic structures and valley property

**Figure 2:**
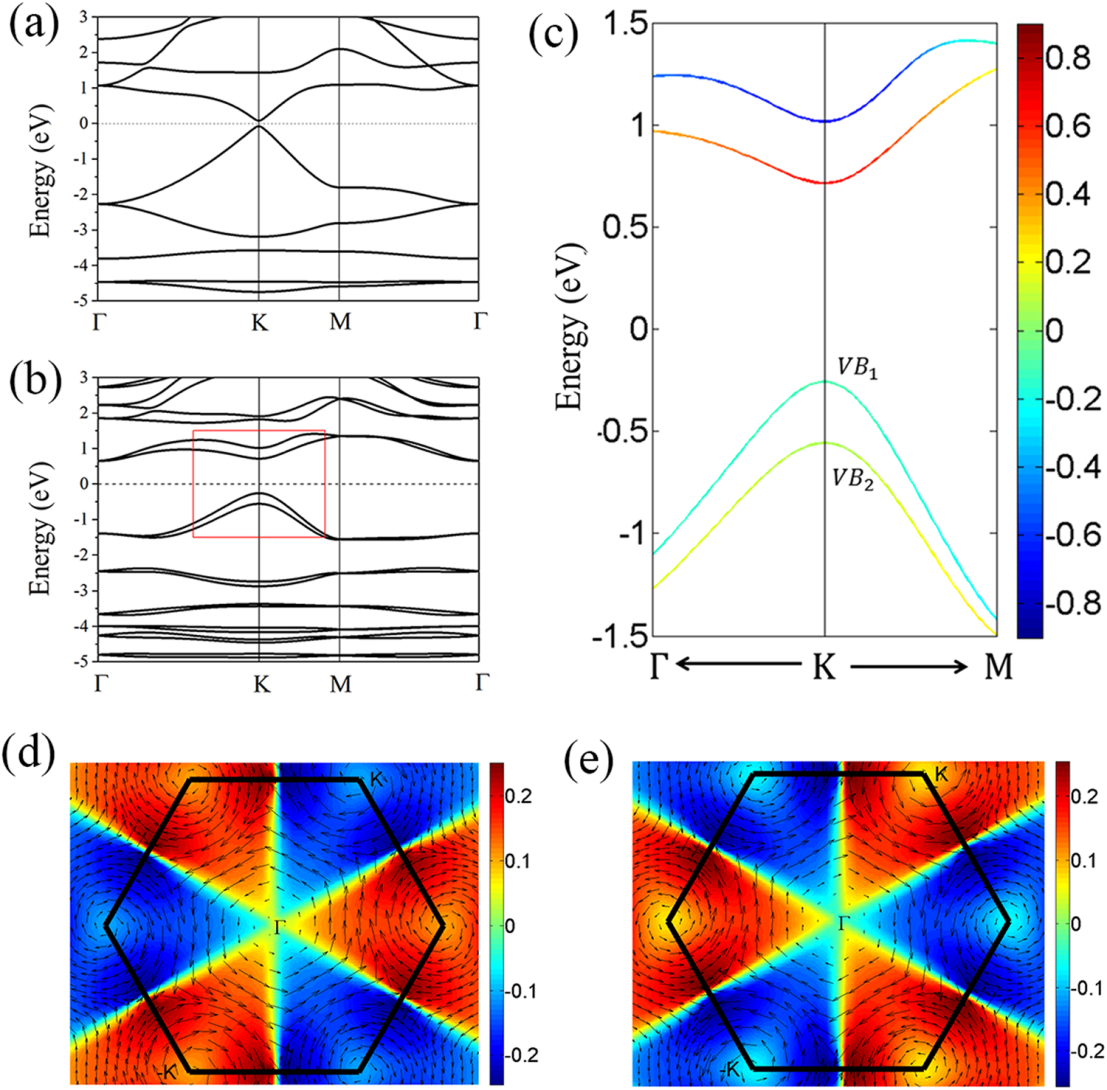
The electronic structure of Bi_2_HF nanosheet. Bands (**a**) without SOC and (**b**) with SOC; (**c**) is the zoom-in of the red box in (**b**), where the color scale refers to as the out-of-plane *S*_*z*_ spin polarization; (**d**) and (**e**) are spin textures of the first and second spin-splitted valence bands, respectively. The arrows denote the in-plane spin direction while the colors indicate the out-of-plane spin direction.

Chemically hydrogenated and fluorinated functionalizations are efficient approaches to change and modify the electronic structures of 2D materials. For instance, the zero band gap of graphene can be tuned to be an insulator when decorated with hydrogen or fluorine^23^ and the functionalized Sb (111) monolayers are proposed to realize the quantum spin quantum anomalous Hall effect^38,39^. The electronic band structures and spin textures of Bi_2_HF nanosheet were calculated. In the absence of SOC, the band structure of Bi_2_HF nanosheet is shown in Fig. 2(a). It suggests that Bi_2_HF nanosheet is a narrow-gap semiconductor with a direct band gap of 0.16 eV at *K* point. Compared to the fully hydrogenated or fluorinated Bi monolayer with a zero band gap in the absence of SOC, this finite band gap of Bi_2_HF nanosheet is due to the noncentrosymmetric structure. In other words, the intrinsic electric field provided by electronegativity difference between hydrogen and fluorine at opposite sides of bismuth in Bi_2_HF nanosheet induces the band gap opening. The physical mechanism is similar to the situation of bilayer graphene^40,41^ and other low dimensional materials^42,43^ under the external electric field.

The band structure of Bi_2_HF nanosheet with SOC is shown in Fig. 2(b), and now each band is splitted by the SOC owing to the noncentrosymmetric structure. The SOC inverses the valence band (VB) and conduction band (CB) and opens a 0.97 eV band gap at K point. The band evolution with the SOC strength changing from 0 to 100% is shown in Fig. S3. When the strength of SOC is set to be 15%, the valence band touches with the conduction band on Fermi level and forms a Dirac point at *K* point. With the continuous increasing of SOC strength, the Dirac point will be gapped and then the band inversion occurs. When the SOC is up to 100%, the band gap is enlarged to 0.97 eV at *K* point. What is more, there exists Zeeman-type spin splittings for the VBs and CBs at *K* valley and the Zeeman-type gaps are 0.3 eV for VBs and CBs. This intrinsic spin splitting gap is even larger than the value 0.27 eV of multilayer WSe_2_ under external electric field^44^. To understand the large spin-split band gaps of VBs and CBs, the projected band structure with p_*x*_ and p_*y*_ orbitals of Bi has been calculated and shown in Fig. S4. The VBs and CBs are dominated by p_*x*_ and p_*y*_ orbitals of Bi and the large spin-split in VBs and CBs is due to the strong SOC from p orbitals of Bi element. Moreover, we used HSE06 functional to calculate the band structures both without and with SOC as shown in Fig. S5. The bandgaps of Bi_2_HF at HSE06 level are 0.15 eV and 1.3 eV for without and with SOC cases, respectively.

The calculated spin textures of the two spin-splitted VBs for the whole BZ are shown in Fig. 2(d,e). One can see the typical Rashba-type spin pattern around the *K* and −*K* valleys with the in-plane spin components rotating clockwise or counterclockwise in spin-splitted VBs. For a single VB, the out-of-plane spin components show opposite polarizations at time-reversed valleys and the in-plane spin components display the same chirality at *K* and −*K* points. Comparing the spin textures of two spin-split VBs, we find that both the Rashba-like chirality and valley-dependent moment are opposite for the spin-splitted VBs. A similar behavior is found for the spin-splitted CBs.

### Effective Hamiltonian model and Berry curvature

To illustrate the origin of the spin texture and valley property, and their interplay with the intrinsic polarization of Bi_2_HF nanosheet, we derive an effective Hamiltonian model of Bi_2_HF nanosheet. Based on the first-principles calculations, around the *K* and −*K* points at Fermi level, the low-energy band structure is dominated by p_*x*_ and p_*y*_ orbitals from Bi atoms (see Fig. S3). So we write the symmetrized basis functions as $|{\varphi }_{1}\rangle =-\,\frac{1}{2}({{\rm{p}}}_{{\rm{x}}}^{{\rm{A}}}+{i{\rm{\tau }}}_{{\rm{z}}}{{\rm{p}}}_{{\rm{y}}}^{{\rm{A}}}),|{\varphi }_{2}\rangle =\frac{1}{2}({{\rm{p}}}_{{\rm{x}}}^{{\rm{B}}}-{i{\rm{\tau }}}_{{\rm{z}}}{{\rm{p}}}_{{\rm{y}}}^{{\rm{B}}})$, with the *τ*_*z*_ representing the valley degree of freedom and A and B are two sites in primitive cell. In the absence of SOC, the low-energy effective Hamiltonian around the *K* point can be written as

2 $${{H}}_{{K}}=\hslash {v}_{F}(\tau {k}_{x}{\sigma }_{x}-{k}_{y}{\sigma }_{y})+m{\sigma }_{z}$$

where the Pauli matrix *σ*_*i *_(*i* = *x*, *y*, *z*) denotes the orbital degree of freedom, *τ* = ±1 represents the valley degree of freedom of *K* and −*K*, the last term describes the staggered sublattice potential induced by inversion symmetry breaking. Compared to the case of graphene^9^, hydrogenated or fluorinated bismuth monolayer^27^, the additional mass term leads to an intrinsic band gap in the energy spectrum, which separates the Dirac points at *K* and −*K*. It is consistent with band structures obtained from the first-principles calculations.

When the SOC is turned on, additional terms appear in the effective Hamiltonian, namely,

3 $${{H}}_{{K}}^{{\rm{soc}}}=\tau ({{\rm{\lambda }}}_{{\rm{so}}}^{+}{{\rm{\sigma }}}_{z}+{{\rm{\lambda }}}_{{\rm{so}}}^{-}){s}_{z}+({{\rm{\lambda }}}_{{\rm{R}}}^{+}{\sigma }_{z}+{{\rm{\lambda }}}_{{\rm{R}}}^{-})\,({k}_{x}{s}_{y}-{k}_{y}{s}_{x})$$

where the Pauli matrix *s*_*i *_(*i* = *x*, *y*, *z*) denotes the spin degree of freedom, ${{\rm{\lambda }}}_{{\rm{so}}}^{\pm }=\frac{1}{2}({{\rm{\lambda }}}_{so}^{{\rm{A}}}\pm {{\rm{\lambda }}}_{{\rm{so}}}^{{\rm{B}}})$ and ${{\rm{\lambda }}}_{{\rm{R}}}^{\pm }=\frac{1}{2}({{\rm{\lambda }}}_{{\rm{R}}}^{{\rm{A}}}\pm {{\rm{\lambda }}}_{{\rm{R}}}^{{\rm{B}}})$. The first term represents the effective spin orbit interaction and ${{\rm{\lambda }}}_{{\rm{so}}}^{A/B}$ is undetermined material-dependent parameter arising from the interplay of SOC, local orbital energy and hopping integrals. The second term represents the Rashba interaction and ${{\rm{\lambda }}}_{{\rm{R}}}^{A/B}$ is a complex material-dependent Rashba parameter. This effective Hamiltonian has the same form with binary III-V monolayer^45^. In the case of ${{\rm{\lambda }}}_{{\rm{so}}}^{-}={{\rm{\lambda }}}_{{\rm{R}}}^{-}=0$, one can recover the full hydrogenated or fluorinated bismuth^46^.

In the first spin-orbit interaction term, the inequivalent A and B sites owing to the noncentrosymmetric structure of Bi_2_HF nanosheet will introduce a non-zero term ${{\rm{\tau }}{\rm{\lambda }}}_{so}^{+}{\sigma }_{z}$ and this term can be regarded as an effective Zeeman-like valley-dependent magnetic field which removes the spin-degeneracy without mixing spin-up and spin-down states. As a result, the spin-splitted VBs and CBs and a net out-of-plane spin polarization at *K* valleys are present (as shown in Fig. 2(b,c)). The second Rashba term induces an in-plane circularly rotating spin-texture around each *K* valley, with opposite charities for spin split VBs (CBs). This is typical Rashba-like behavior, which appears to be valley-independent, in agreement with our first-principles calculations.

**Figure 3:**
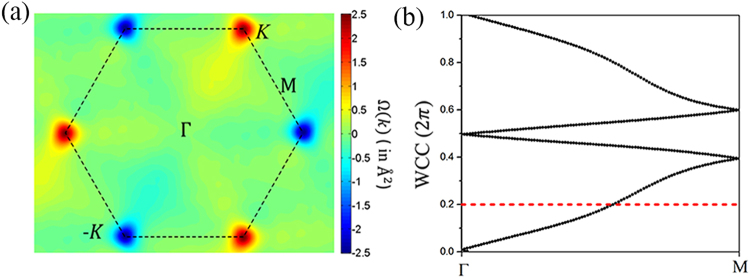
(**a**) The out-of-plane Berry curvature on *k*_*z*_ = 0 plane in the momentum space of Bi_2_HF nanosheet. (**b**) The tracking of the evolution of the WCC between two time-reversal invariant momenta (TRIM) points in the reciprocal space *k*_*z*_ = 0 plane. The dashed red line is a reference line to track the number of Wannier center pair.

To further illustrate the valley properties of Bi_2_HF nanosheet, the Berry curvature has been calculated using the usual linear response Kubo-like formula^47^

4 $${\rm{\Omega }}(k)=\sum _{n}\,{f}_{n}{{\rm{\Omega }}}_{n}(k)$$

5 $${{\rm{\Omega }}}_{n}(k)=-\,2Im\sum _{m\ne n}\frac{\langle {u}_{nk}|{v}_{x}|{u}_{mk}\rangle \langle {u}_{mk}|{v}_{y}|{u}_{nk}\rangle }{{({E}_{mk}-{E}_{nk})}^{2}}$$

where *f*_*n*_ is the Fermi distribution function, *v*_*x*,*y*_ is the velocity operator, and *u*_*nk*_ is the periodic part eigenvector with eigenvalue *E*_*nk*_ of the Fourier transformed Wannier Hamiltonian as calculated by projecting the density functional theory (DFT) Hamiltonian onto a Wannier basis^48^. The calculated out-of-plane Berry curvature of Bi_2_HF nanosheet is shown in Fig. 3(a), where the opposite signs at *K* and −*K* valleys are found.

### Quantum spin Hall Effect and helical edge states

Now we focus on the topological property and QSHE in Bi_2_HF nanosheet. The Bi element is well known for its strong SOC that can drive and stabilize the topological nontrivial states. A variety of Bi-related materials with a large band gap have been proposed and predicted as room temperature QSH insulators^16,17,27,28,49^. As mentioned above, the strong SOC in Bi_2_HF nanosheet inverses the valence and conduction bands at *K* point. This is an important feature of topological insulators. The band topology of Bi_2_HF nanosheet can be characterized by the *Z*_2_ invariant. *Z*_2_ = 1 characterizes a nontrivial band topology (corresponding to a QSH insulator), whereas *Z*_2_ = 0 represents a trivial band topology. To confirm that the Bi_2_HF nanosheet is a topological insulator, we need to calculate its *Z*_2_ invariant. Since the Bi_2_HF nanosheet is a noncentrosymmetric structure, the parity method of Fu-Kane formula^50^ does not work here. Instead, the Wilson loop method should be calculated by Wannier charge centers (WCC) to confirm the topological invariant of Bi_2_HF nanosheet. As shown in Fig. 3(b), the evolution lines of WCC between two time-reversal invariant momenta (TRIM) of the BZ cross the arbitrary reference line odd number of times in *k*_*z*_ = 0 plane, indicating the topological invariant *Z*_2_ = 1 and Bi_2_HF nanosheet is indeed a 2D topological insulator. We also confirm the topological nontrivial feature of Bi_2_HF by using the HSE06 functional (See Fig. S5(c)).

**Figure 4:**
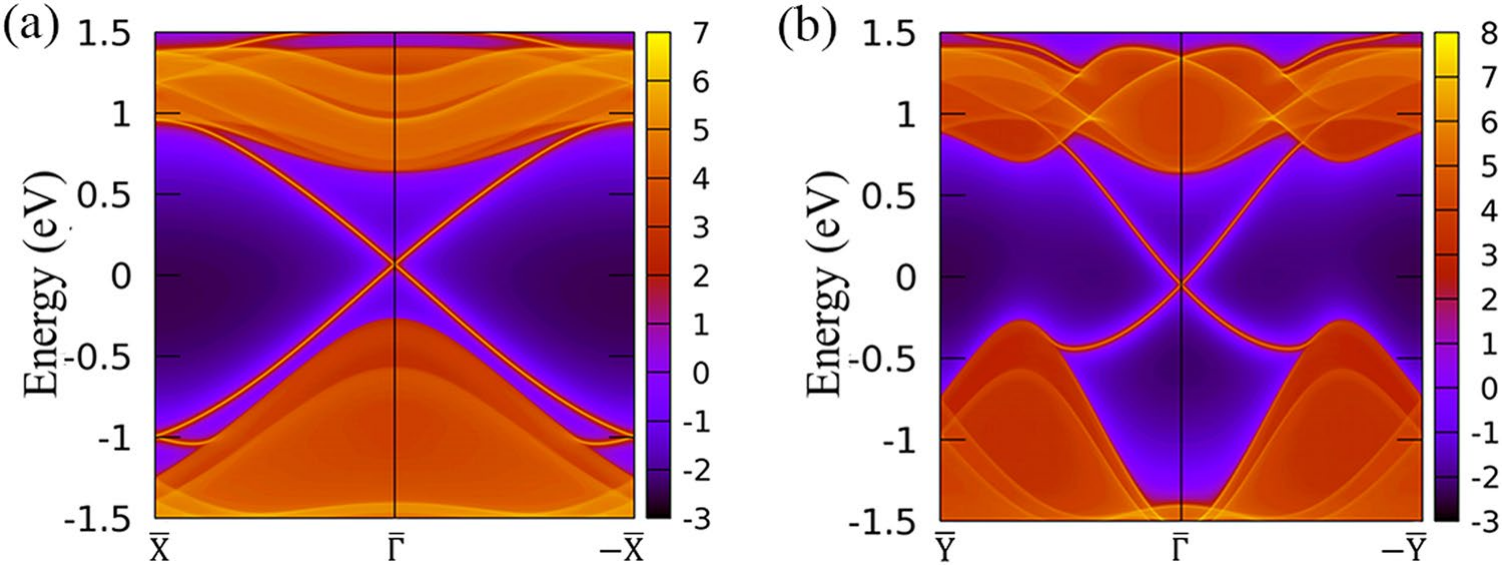
The local density of states of edge states for Bi_2_HF nanosheet with (**a**) the armchair edge and with (**b**) the zigzag edge.

The helical metallic edge state is a hallmark of the 2D topological insulator. To justify the topological helical metallic edge state of Bi_2_HF nanosheet, the local density states of two typical semi-infinite edges, namely armchair and zigzag edges, have been calculated using a recursive Green’s function method. As shown in Fig. 4(a,b), it is clear that the helical edge states disperse in the bulk band gap and cross linearly at $\bar{{\rm{\Gamma }}}$ point. The band structures of the armchair and zigzag nanoribbons with 20 unitcells are calculated using tight-binding method and are shown in the Fig. S6. The results are consistent with our band structures from Green’s function calculations. Note that the left and right edges of the zigzag nanoribbon have different terminations, but the armchair nanoribbon has the same termination for left and right edges. So one can see two Dirac cones at $\bar{{\rm{\Gamma }}}$ in Fig. S6(b) due to the different chemical potential of left and right edges in the zigzag nanoribbon. These features further prove the nontrivial nature of Bi_2_HF nanosheet, which is in agreement with *Z*_2_ calculations. Remarkably, the Dirac points formed by the helical edge states are very close to Fermi level, which is important for practical spintronics application. Moreover, the giant bulk gap 0.97 eV of Bi_2_HF nanosheet can stabilize the edge states against the thermally activated carriers, which is beneficial for realizing room-temperature QSHE in Bi_2_HF nanosheet. To further confirm the robustness of topological properties of Bi_2_HF nanosheet with respect to temperature, we calculate *Z*_2_ invariant and topological metallic edge state using 3 × 3 supercell of Bi_2_HF after 6 ps AIMD at 300 K. The evolution of WCC of AIMD supercell of Bi_2_HF nanosheet shows stable topological nontrivial *Z*_2_, and the helical metallic edge state of semi-infinite edge of Bi_2_HF supercell is seen at 300 K as shown in Fig. S7.

## Conclusions

In summary, we propose from first-principles that a hydrofluorinated bismuth nanosheet is a 2D topological insulator with a giant bulk band gap of 0.97 eV and it is expected to achieve the room-temperature QSHE. The band topology of Bi_2_HF nanosheet has been confirmed by Z_2_ topological invariant using Wilson loop method and the recursive Green’s function calculations also reveal the helical metallic edge states in Bi_2_HF nanoribbon. The phonon dispersion and AIMD calculations show Bi_2_HF nanosheet structure is dynamically stable and robust at room temperature. Furthermore, the co-decoration of H and F elements on opposite sides of Bi monolayer induces an intrinsic out-of-plane electric field and thus results in the valley-related properties. The interplay between such intrinsic polarization and SOC generates in-plane Rashba-type spin texture and out-of-plane spin splitting of band structure in Bi_2_HF nanosheet. The QSHE and valley polarization in Bi_2_HF nanosheet may find promising applications in future spintronics.

## Methods

To investigate the valley and topological properties of Bi_2_HF nanosheet, we carried out first-principles calculations of DFT as implemented with plane-wave basis set in Vienna *ab initio* simulation package (VASP)^51^. The exchange correlation interaction was treated within the generalized gradient approximation (GGA)^52^ parameterized by the Perdew, Burke, and Ernzerhof (PBE). The energy cutoff of 500 eV was set in all the calculations and the structures are well relaxed until the Hellmann-Feynman forces on all atoms are less than 0.005 eV/Å. A Monkhorst-Pack grid with 17 × 17 × 1 k-points was used for BZ integration. SOC was taken into account self-consistently in terms of the second vibrational procedure^53^. To overcome the underestimation of bandgap by the PBE functional, we used HSE06 hybrid functional to confirm our results. A large vacuum region of 20 Å was applied along *z* direction to minimize the interaction between two slabs from the periodic boundary condition. To check the dynamic stability, we present the phonon dispersion of Bi_2_HF by employing the PHONOPY code^54^ through the DFPT approach^35^. To explore the edge states of Bi_2_HF nanosheet, the effective Hamiltonian was constructed by using maximally localized Wannier function (MLWF) for p orbitals of Bi as implemented in the Wannier90 package^48^. We used the recursive Green’s function method^55^ based on MLWF to calculate the local density state of infinite nanosheet. The imaginary part of the surface Green’s function is related to the local density of states (LDOS), from which we can obtain the edge states. Regarding the absence of inversion center in Bi_2_HF, the Z_2_ invariant was computed by tracing the WCC using the non-Abelian Berry connection^56,57^ as implemented in the Z2pack software^58^.

## Electronic supplementary material

Supplementary Information
