# Supplementary material for: Spin valley and giant quantum spin Hall gap of hydrofluorinated bismuth nanosheet
Source: Sci Rep. 2018 May 9;8:7436. doi: 10.1038/s41598-018-25478-6 (PMC5943254; doi:10.1038/s41598-018-25478-6)
Supplement: Supplementary file 1 — Supplementary Information [file 41598_2018_25478_MOESM1_ESM.pdf]

Supplementary Information of

Spin valley and giant quantum spin Hall gap of hydrofluorinated bismuth nanosheet

Heng Gao[1], Wei Wu[1], Tao Hu[1], Alessandro Stroppa[2], Xinran Wang[3], Baigeng Wang[4], Feng Miao[4], Wei Ren*[1]

1 Department of physics, Materials Genome Institute, and International Centre for Quantum and Molecular Structures, Shanghai University, 200444 Shanghai, China

2 Consiglio Nazionale delle Ricerche (CNR-SPIN), Via Vetoio, I-67010 L'Aquila, Italy

3 School of Electronic Science and Engineering, and Collaborative Innovation Center of Advanced Microstructures Nanjing University, 210093 Nanjing, China

4 National Laboratory of Solid State Microstructures, School of Physics, Collaborative Innovation Center of Advanced Microstructures, Nanjing University, 210093 Nanjing, China

*renwei@shu.edu.cn

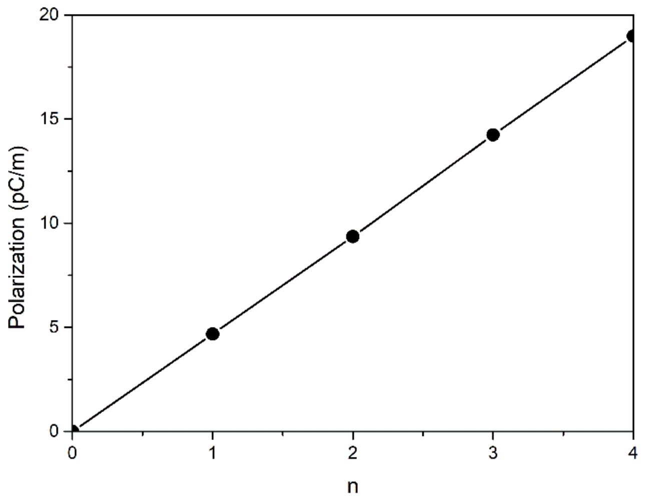

Figure S1. Polarization linearly increases as a function of n from 0 (i.e., the nonpolar structure of fully hydrogenated Bi) to 4 (i.e., the polar structure of hydrofluorinated Bi). Here n is the number of F atoms in the $2 \times 2 \times 1$ supercell.

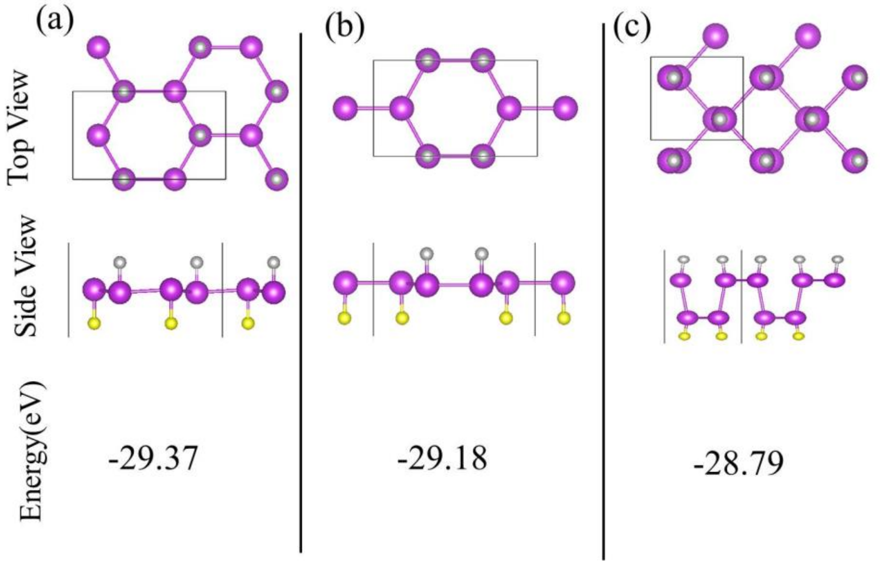

Figure S2. Geometric configuration and total energy of (a) chair (b) boat (c) black phosphorene-like Bi_2HF. Rectangle boxes represent conventional unit cells.

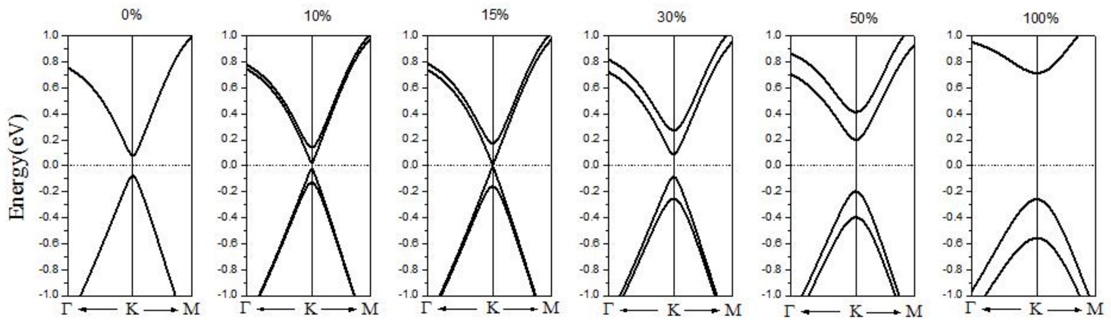

Figure S3. The band structure evolution and band inversion with the spin-orbit coupling (SOC) strength increasing from 0 to 100%. When the SOC strength increases to 15%, the valence band touches with the conduction band at *K* point.

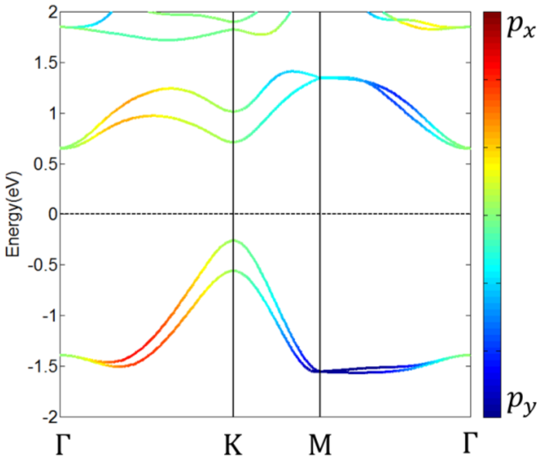

Figure S4. The projected band structure with p_x and p_y orbitals of Bi, where the red and blue colors denote p_x and p_y orbitals, respectively.

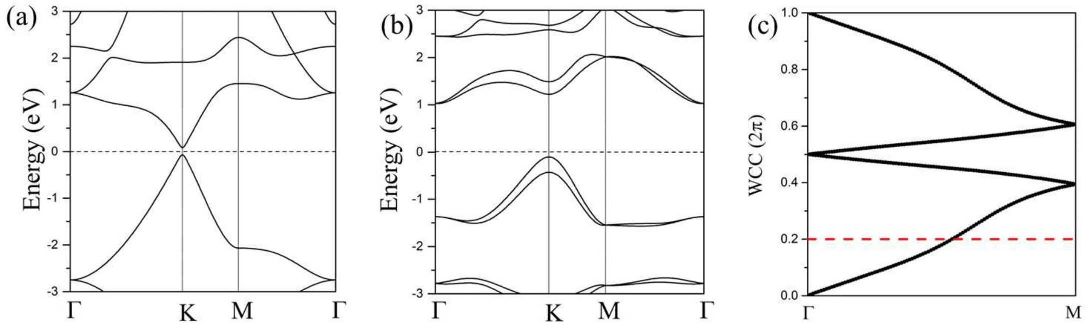

Figure S5. The calculated band structures (a) without and (b) with SOC, and Z_2 invariant for Bi_2HF using HSE06 functional. (c) Evolution of Wannier charge center.

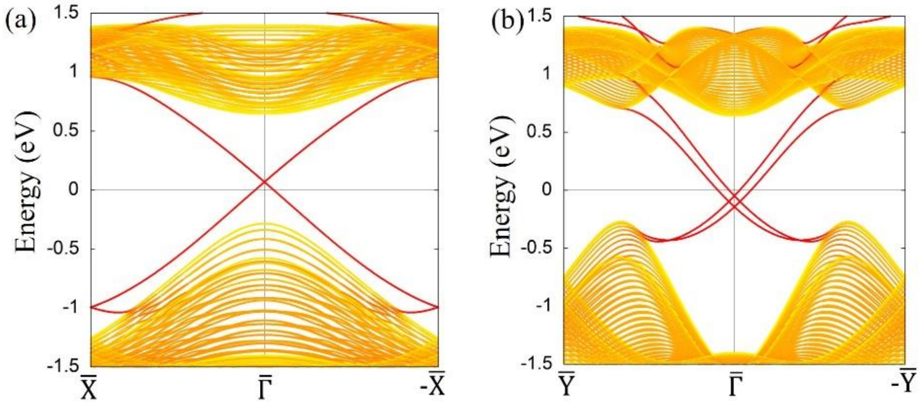

Figure S6. The calculated band structures of (a) armchair (b) zigzag Bi_2HF nanoribbon with 20 unitcells from the tight-binding method.

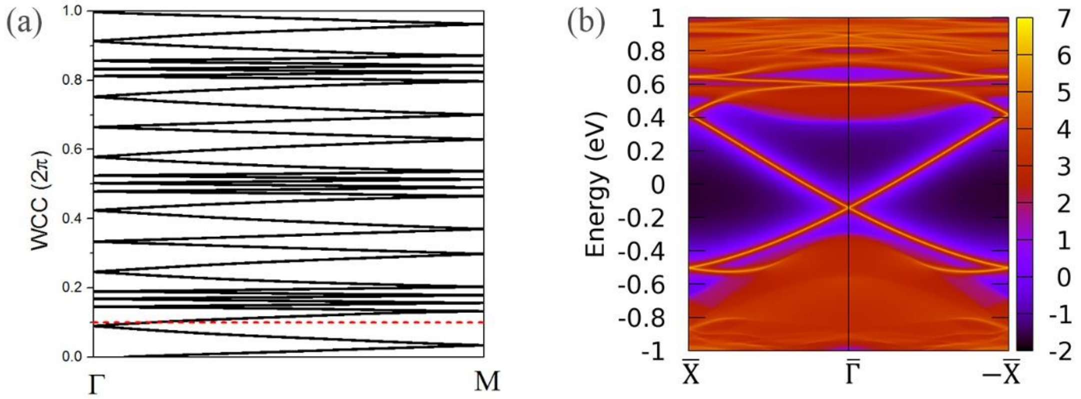

Figure S7. (a) Evolution of Wannier charge center (WCC) and (b) band structure of edge states of Bi_2HF nanosheet supercell after 6 ps in *ab initio* molecular dynamics (AIMD) simulation at 300 K.
